# Clarithromycin Suppresses Apple Proliferation Phytoplasma in Explant Cultures

**DOI:** 10.3390/plants12223820

**Published:** 2023-11-10

**Authors:** Matěj Semerák, Jiří Sedlák, Radek Čmejla

**Affiliations:** Research and Breeding Institute of Pomology Holovousy Ltd., 50801 Holovousy, Czech Republic

**Keywords:** apple, in vitro, sanitation, phytoplasma, proliferation, antibiotics, clarithromycin

## Abstract

Apple proliferation, caused by ‘*Candidatus* Phytoplasma mali’, is one of the most important economic threats in the field of apple production. Especially at a young age, infected trees can be affected by excessive bud proliferation and general decline. The fruit quality is also significantly reduced by this disease. To investigate treatment options, we applied a clarithromycin chemotherapy to infected in vitro cultures of ‘Golden Delicious’. With increasing concentrations of clarithromycin in the media, the phytoplasma load decreased rapidly after one month of treatment, but phytotoxicity led to a pronounced mortality at 40 mg/L, which was the highest dose used in our experiment. Out of 45 initial explants, we obtained one negative mericlone and two mericlones with a concentration of phytoplasma DNA at the detection limit of PCR. The culture propagated from the mericlone that tested negative remained phytoplasma-free after 18 months of subculturing. Our results suggest the applicability of macrolide antibiotics against phytoplasma infections in vitro; however, it might be challenging to find the threshold zone where the concentration is sufficient for pathogen elimination, but not lethal for the plant material of different cultivars.

## 1. Introduction

Phytoplasmas (kingdom: Bacteria; phylum: Mycoplasmatota) are plant-phloem- or insect-haemolymph-inhabiting vector-borne and graft-transmissible prokaryotes without a cell wall that, unlike other bacteria, cannot be cultured on artificial nutrient media and are restricted to the phloem part of the plant [1,2,3]. They can be transmitted by infected vegetative plant material or by parasitic plants [4,5]. Historical records dating back to ancient China describe the typical symptoms of a phytoplasma infection in green-flowered peonies. However, unlike other groups of microorganisms, they were discovered and scientifically described as mycoplasma-like organisms relatively late, only in 1967, in ultrathin electron microscopic sections of phloem tissues from infected mulberry and aster plants showing typical symptoms, such as flower abnormalities, yellowing, and witches’ brooms [6]. Until then, these systemic and persistent symptoms typical of phytoplasmas were considered to be a manifestation of a viral infection [7,8].

Phytoplasmas are found only in vascular bundles in the sieve tube elements, and especially in woody plants, they are very unevenly distributed in different parts of the plant [9]. According to the current biological nomenclature, phytoplasmas belong to the class *Mollicutes* and the genus ‘*Candidatus* Phytoplasma’. Woody fruit plants of the family *Rosaceae* may be seriously affected by phytoplasmas of the apple proliferation group (AP 16SrX group) [10]. The quarantine pathogenic organism ‘*Candidatus* Phytoplasma mali’ (‘*Ca*. P. mali’) is the causal agent of apple proliferation (AP). AP phytoplasma causes considerable economic damage annually in the nursery sector and in Central European apple plantations [10].

The pathogen is reliably detected in symptomatic apple trees. However, it can also occur in the form of latent infections, where a reliable diagnosis can be problematic. The bacterium adsorbs some of the host’s metabolic substances and clogs the conductive tissues, leading to the manifestation of the infection through a number of specific and non-specific symptoms. Among the most prominent specific symptoms are premature shoot growth; the formation of a shoot bundle, usually called a witches’ broom; and a marked enlargement of the stipules at the base of the petiole [11,12]. The bacterium also triggers physiological changes such as the emission of volatile organic compounds [13]. Considering the economic damage, one of the key problems of AP infections is the negative impact on fruit. The fruit from infected host apple trees is significantly smaller and has a poorer flavour quality [12].

AP phytoplasma is a systemic infection, and affected trees cannot be effectively treated with conventional plant protection products in a permanent location under field conditions. Attempts to apply tetracycline antibiotics on phytoplasma-affected fruit trees by transfusions or spraying have been published [14]. However, such approaches are unacceptable for production plantations, and they are also legislatively prohibited in European countries. The control of phytoplasma diseases in orchards mostly depends on indirect methods, such as the use of insecticides against their insect vectors from the *Cacopsylla* genus.

In the market area of nursery plant material production, the propagation of uninfected initial plants at the beginning of the propagation cycle is a very important and key step within the certification scheme. In general, not much work has been published so far on phytoplasma eradication for the initial elite nursery material of temperate woody fruit species, although there are some promising results for hot water thermotherapy applied to dormant grafts [15,16].

Biotechnological methods using in vitro cultures grown under protected and controlled laboratory conditions offer a future perspective for improving the efficiency of sanitation from systemic internal infections of the phytoplasma type. If the first steps succeed, i.e., the surface sterilization of initial explants and the subsequent induction of micropropagation, the established actively growing and multiplying in vitro cultures represent the optimal starting platform for subsequent therapeutic procedures. In vitro cultures can be propagated in hundreds of actively growing shoot tips on several square metres in a cultivation room throughout the year, regardless of the vegetation season. In the sterile environment of closed laboratory vessels, the risk of infection with microbial pathogens is minimized and contact with potential vector organisms of the *Cacopsylla* type is excluded. In addition, the risk of reinfection of already sanitated material in the in vitro propagation and rooting phase is virtually eliminated.

The in vitro approach also offers several sanitation options. AP phytoplasma is not a pathogen that can be reliably eliminated by a simple in vitro transfer or gradually suppressed by subculturing. It persists in the micropropagated material for years [17], and thus, other advanced techniques are required.

Apart from hot water treatments applied on grafts, thermotherapy can be coupled with the establishment of in vitro cultures as well. This method was used for the elimination of phytoplasmas from raspberries and blackberries [18] and for plants from the *Malus*, *Pyrus*, and *Prunus* genera [19]. The elimination of AP phytoplasma, apple chlorotic leaf spot virus, and apple mosaic virus using in vitro cultures and cryo-knife therapy was reported in our previous work [20]. In vitro stock shoots of Chinese jujube were sanitated from ‘*Ca.* Phytoplasma ziziphi’ through droplet vitrification cryopreservation [21]. As for antibiotic treatments against phytoplasmas, experiments with tetracycline-type substances are most often mentioned. It was reported that treatments with tetracycline antibiotics suppressed phytoplasma propagation in infected plants cultured in vitro, but high concentrations of antibiotics caused tissue damage. Tetracyclines had a bacteriostatic effect on phytoplasmas in treated plants, but the symptoms usually reappeared after transfer to an antibiotic-free medium [22,23,24,25].

Other types of antibiotics have also been used against phytoplasmas in previous experiments. Tanno et al. [26] evaluated 40 antimicrobial compounds against ‘*Ca*. Phytoplasma asteris’, and they found that, besides tetracyclines, floroquinolones, macrolides, phenicols, rifamycins, and pyrimidine antagonists also significantly decreased the phytoplasma abundance. Phytohormone-like substances, namely cytokinins of the kinetin type [27,28] or auxins [29,30], have also been tested as antimicrobial candidates against phytoplasmas with unsatisfactory final results in terms of recovery.

This paper reports the results of the use of clarithromycin, the prospective macrolid antibiotic, and ciprofloxacin, a member of the floroquinolone family, for AP control in the reference apple cultivar ‘Golden Delicious’.

## 2. Results

**Figure 1:**
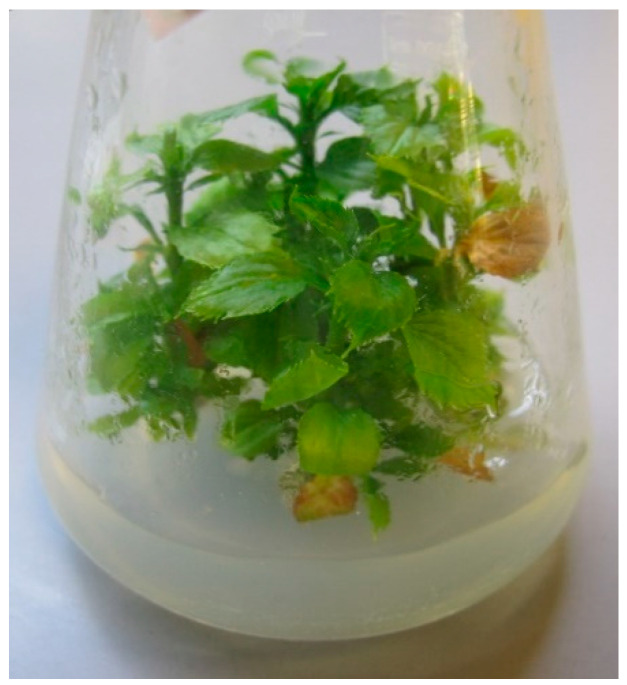
Explant culture before chemotherapy.

AP phytoplasma DNA was found in all in vitro control samples of ‘Golden Delicious’ collected at the start of the chemotherapy treatments. The concentrations reached half of the level previously detected in the mother plant, but they remained highly positive with Ct values of around 16 (Table 1). Despite this fact, the material did not show any remarkable symptoms (Figure 1).

### 2.1. Phytotoxicity

**Figure 2:**
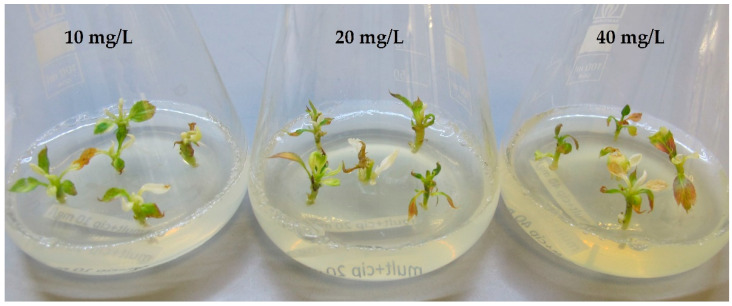
Phytotoxicity of ciprofloxacin after one month at concentrations of 10, 20, and 40 mg/L.

**Figure 3:**
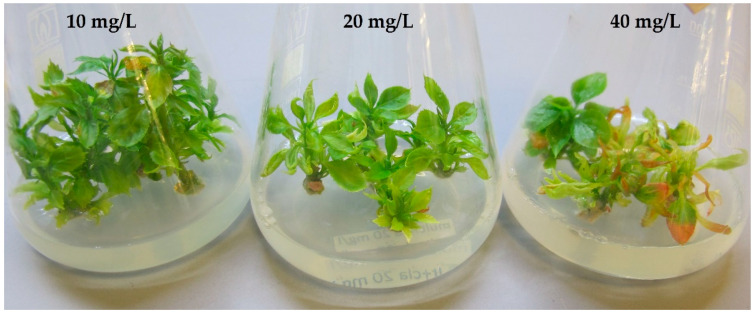
Phytotoxicity of clarithromycin after one month at concentrations of 10, 20, and 40 mg/L.

In all ‘Golden Delicious’ cultures treated with ciprofloxacin, the phytotoxic effect became apparent early in the culture development. The explants failed to grow and showed leaf chlorosis and deformations (Figure 2). After one month of cultivation, the shoot tips became etiolated or disintegrated. In contrast, the application of clarithromycin resulted in less severe phytotoxicity symptoms (Figure 3). All 15 mericlones on the media containing 10 mg/L clarithromycin survived, although mild symptoms of phytotoxicity such as slow growth were evident. At a concentration of 20 mg/L, 1 mericlone died, while the other 14 were moderately affected, showing stunted growth, mild chlorosis, and leaf deformation. The 40 mg/L variant showed high toxicity, and 12 out of the 15 initial mericlones died.

### 2.2. Efficiency of Chemotherapy

**Figure 4:**
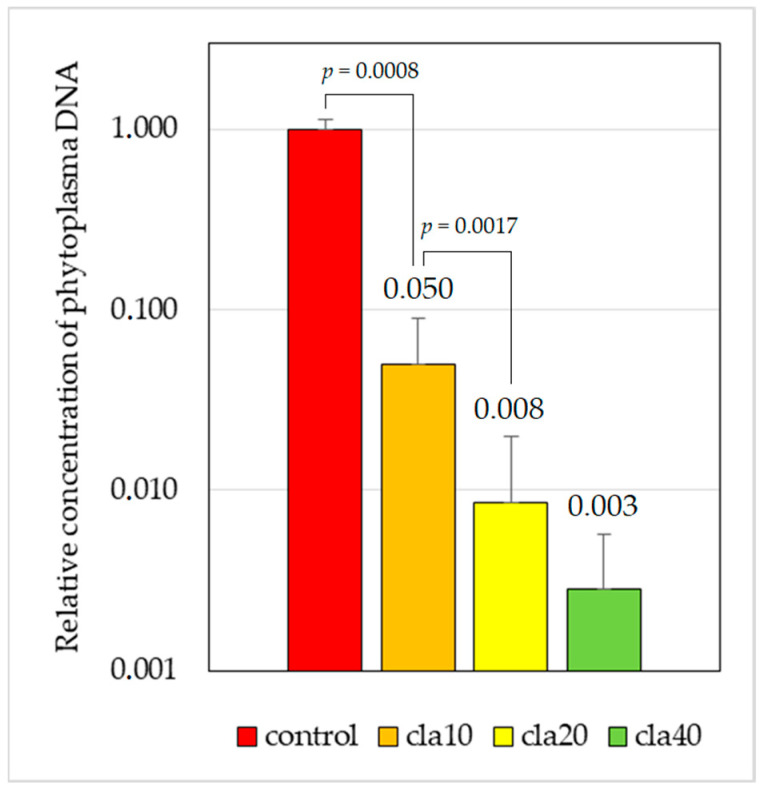
Average relative concentration of AP phytoplasma DNA after 1 month of treatment on media with clarithromycin. Phytoplasma load in initial in vitro cultures was set to 1. Y-axis is in logarithmic scale. Error bars depict standard deviation. cla10 = medium with 10 mg/L; cla20 = medium with 20 mg/L; cla 40 = medium with 40 mg/L of clarithromycin.

The results of the tests performed on the 32 surviving mericlones after chemotherapy are shown in Table 1 and Figure 4. They indicate that the elimination efficiency increased with higher concentrations of clarithromycin. The differences between the untreated control and the treatments with 10 and 20 mg/L were statistically significant (*p*-values < 0.01). In the case of the treatment with 40 mg/L, statistical analyses could not be carried out due to a low number of samples, resulting from a high lethality. Complete eradication was demonstrated with one mericlone treated with 20 mg/L, and a particularly high pathogen Ct value of about 34 was obtained for two other mericlones (one on the medium containing 20 mg/L and one on the medium containing 40 mg/L).

**Figure 5:**
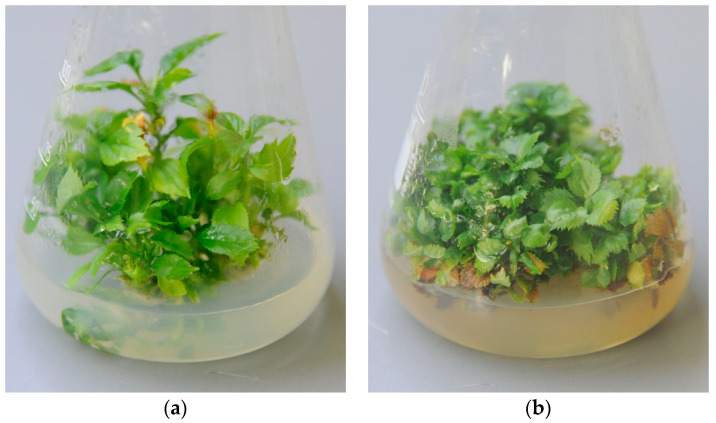
Culture derived from AP-negative ‘Golden Delicious’ mericlone 18 months after chemotherapy (**a**), compared to untreated initial material kept in parallel (**b**).

After performing the PCR tests, we transferred the negative-tested mericlone for regeneration to the medium without antibiotics under the same conditions used for multiplication. This explant showed vigorous growth and produced healthy-looking lateral shoots in the course of further subcultures. At the same time, the untreated culture, which was being subcultured in parallel, declined slightly. The explants showed an abnormally high multiplication compared to the original starting culture, but their longitudinal growth slowed down and the leaf size gradually decreased. Over the following months, the visible difference in the chemotherapeutically treated material gradually became more pronounced (Figure 5a,b).

Noticing this gradual deterioration in health, we re-determined the phytoplasma load in the untreated control, namely 18 months after the initial tests. As in the initial tests, we used four random samples. The results confirmed that, without therapy, phytoplasmas accumulated in the cultures. On average, their concentration showed a 2.4-fold increase compared to the samples analysed as controls at the beginning of our experiments. At the same time, we also determined the phytoplasma presence in one mixed sample obtained from the healthy-looking material originating from the mericlone cla20 (10), which tested negative immediately after the chemotherapy. The sample tested negative (Table 2).

## 3. Discussion

Plants infected with phytoplasmas show symptoms indicating a profound disturbance in the normal balance of growth regulators [2,9]. Therefore, in vitro chemotherapeutic treatments must take into account that the phytoplasma-infected plant is not only affected by the artificially added plant growth regulators, but also by this significant change in its otherwise natural phytohormonal regulation. Phytoplasmas can also reach consistently higher concentrations in long-term micropropagated explants than in samples from field-grown plants, which can make them difficult to eradicate under in vitro conditions [20,25]. On the other hand, we found in our previous work with plants from the *Maloideae* subfamily that phytoplasmas ‘*Ca.* P. mali’ and ‘*Ca*. P. pyri’ can be eliminated after the extirpation and sterilisation of the initial explants, followed by an in vitro transfer and cultivation. We observed this result in the apple cultivars ‘Panenské zlepšené’ and ‘Virginia Crab’ [20], and the pears ‘Williams’, ‘Conference’, ‘Bohemica’, and ‘Manon’. These established cultures showed vigorous growth and tested PCR-negative for phytoplasmas. We suppose that the success of this sanitation method might depend on the proper timing of material collection from in vivo conditions, because phytoplasmas are known to circulate in plants periodically during the year [31], and on the technique of bud excision.

AP-infected in vitro cultures of ‘Golden Delicious’ show well-defined symptoms of phytoplasma presence, such as stunted growth, proliferation, and reduced leaf size [32]. During the first months after the in vitro transfer of our infected material, we did not observe any striking signs (Figure 1), but further maintenance of the cultures that did not undergo any chemotherapeutic treatments led to the manifestation of the reported symptoms (Figure 5b), suggesting that the prolonged micropropagation of AP-infected explants deteriorates their health status. This is similar to our recent findings that explant cultures of the apple variety ‘Táborita’, infected with AP phytoplasma and two viruses, gradually declined after cryotherapy, which failed to eradicate the pathogens [20].

The repeated PCR testing of long-term maintained untreated material showed that phytoplasmas reached extremely high concentrations in the tissues, which is consistent with previous reports [25] and probably led to the worsening of the symptoms. Based on our observation of these untreated cultures, we also hypothesise that, due to the generally slow growth of micropropagated material, the phytoplasmas could gradually accumulate massively, even in the meristematic areas, causing the progressive slowing of growth and pronounced symptom manifestation. This phenomenon could complicate the sanitation efforts. Therefore, we do not recommend prolonged subcultures of phytoplasma-infected explants (for example, to multiplicate the initial culture to a great extent in order to obtain a larger number of explants for the sanitation process).

The gradual health deterioration of this particular culture may also have been accelerated by a co-infection with apple chlorotic leaf spot virus (ACLSV), which was also found to be very abundant in the untreated in vitro material. The character of the symptoms suggested that the AP phytoplasma was more important, but the contribution of the virus could not be excluded. Synergistic effects of different infections have been observed or suspected in fruit trees [33,34]. In any case, the long-lasting disappearance of a phytoplasma infection in the mericlone cla20 (10) led to a visible improvement in its health state. Macrolide antibiotics target bacterial proteosynthesis by inhibiting the 50S ribosomal subunit [35], but it has also been observed in clinical studies that they can affect viral diseases [36]. Control testing of the phytoplasma-free mericlone cla20 (10) showed that, although the chemotherapy reduced the ACLSV load, it still persisted in high concentrations.

During chemotherapeutic treatment, high concentrations of antibiotics can cause tissue damage [22,23,24]. Compared to chrysanthemum cultures infected with ‘*Ca*. Phytoplasma asteris’, as studied by Tanno et al. [26], ‘Golden Delicious’ in our experiment showed a higher level of tolerance to clarithromycin diluted in the growth medium. While all the chrysanthemum explants died at a concentration of 20 mg/L, some apple cultures survived even at 40 mg/L. This allowed some ‘Golden Delicious’ mericlones to be obtained with a significantly reduced phytoplasma load, whereas no convincing effect of clarithromycin was observed for chrysanthemums at lower concentrations. In contrast, an analogous higher tolerance was not confirmed for ciprofloxacin. Although chrysanthemums survived at 5 mg/L, all apple cultures showed serious deterioration at 10 mg/L, which was the lowest dose we used, and due to the disintegration of shoot tips, it was impossible to regenerate them from the apical meristem. In order to investigate the efficiency of ciprofloxacin in eradicating AP phytoplasma, the concentration in the growth medium would have to be reduced in future research.

The applied clarithromycin treatments led to the considerable suppression of phytoplasmas in our experiment. However, complete sanitation was achieved in one clone only. These results might have been influenced by the fact that the concentration of phytoplasmas in the initial material from the gene bank of microorganisms was extremely high: there was a Ct value of 15.09 for the mother plant, and 15.34–16.86 for the derived cultures. In such a situation, we suggest combining chemotherapy and thermotherapy to suppress the pathogen load.

The high variability within the results of each treatment might have been influenced by the individual stress reaction of each explant to the phytotoxic effect of particular concentrations of antibiotics. In this case, the phytoplasma concentration would be affected by the morphology of the explant and by the presence of polyphenols and their oxidative products. Secondary metabolites such as polyphenols are involved in the reactions to abiotic stress [37], they are known to have antibacterial properties, and they can act synergistically with antibiotics [38]. We consider our work to be a model example of a sanitation procedure, which can be used in laboratories not equipped with a thermotherapy cultivation room. In the case of the routine conservation of fruit genetic resources in gene banks, a lower concentration of phytoplasmas can be expected, because the material would not originate from plants that are intentionally kept as a source of phytoplasma infection. This would likely result in a more straightforward effect of the therapy and a higher efficiency in providing healthy material.

The end result of any therapy should be a healthy in vivo material that can be used in orchards or for nursery production. In the case of in vitro sanitation, this requires a successful ex vitro transfer and repeated health testing in subsequent years. Negative results of these tests should confirm the efficiency of the sanitation and rule out the possibility that the pathogen load was only temporarily suppressed below the detection limit of the test methods used. Apparent remissions of infection may already occur during the in vitro regeneration of the material after therapy [23], but further in vivo testing is needed to detect potential delayed pathogen accumulation after the ex vitro transfer [39].

## 4. Materials and Methods

The experimental explant cultures were established using the material from an in vivo gene bank of microorganisms kept in technical isolation at the Research and Breeding Institute of Pomology in Holovousy. The scions were obtained at the end of dormancy from a ‘Golden Delicious’ plant that had tested positive for AP phytoplasma before the start of this project.

After initial sprouting under laboratory conditions, 10 vegetative buds were excised and sterilised in 0.15% HgCl_2_ for 1 min. After two rinses with sterile demineralised water, the buds were planted on a growth medium in Erlenmeyer flasks.

The medium was prepared according to Murashige and Skoog 1962 [40] with the addition of 4 mg/L of ascorbic acid, 1.5 mg/L of 6-benzylaminopurine, and 0.1 mg/L of indole-3-butyric acid. Sucrose was added at a concentration of 30 g/L. The pH value of the media was adjusted to 5.7. Difco Bacto agar in the amount of 8.5 mg/L was used as the solidifying agent. The flasks containing the medium were autoclaved at 121 °C and 200 kPa for 20 min. In the cultivation room, the temperature was set at 22 ± 2 °C and the photoperiod was set at 12 h.

In the first phase, the planted buds were transferred to fresh media every day, until the browning of the media subsided. The subcultivation interval was then extended to one month. After one year, when a sufficient number of shoots had been obtained, four random samples were taken from the cultures for the PCR detection of the presence of AP phytoplasma. At the same time, a chemotherapeutic treatment was applied.

Media containing clarithromycin or ciprofloxacin were prepared. The medium with a standard composition was autoclaved in glass bottles, and the antibiotics (Cayman Chemical Company) were diluted separately: clarithromycin in dimethyl sulfoxide and ciprofloxacin in water. As the medium cooled, the clarithromycin or ciprofloxacin solution was added through antibacterial filters to reach final concentrations of 10, 20, or 40 mg/L. The media were then transferred to Erlenmeyer flasks. For each concentration of both antibiotics, 15 ‘Golden Delicious’ shoots were planted on the medium. After one month, leaf material taken from each of the surviving mericlones was tested using PCR to determine the presence of AP phytoplasma.

Prior to PCR testing, approximately 100 mg of leaves was ground in liquid nitrogen. The total DNA was isolated from the pulverised samples using an Exgene Plant SV isolation kit (GeneAll Biotechnology, Seoul, Republic of Korea) according to the manufacturer’s instructions. The qPCR 2x Blue Master Mix (Top-Bio, Vestec, Czech Republic) was used for real-time PCR detection. The general reaction conditions were set as follows: final volume—20 µL; final concentration of primers/probes—0.5 µM; and template—2 µL of isolated DNA. The AP phytoplasma detection was performed in a Rotor-Gene Q cycler (Qiagen) using the following temperature profile: denaturation at 94 °C/5 min and cycling 45× (94 °C/20 s, 58 °C/20 s with a fluorescence reading, and 72 °C/20 s).

For the detection of AP phytoplasma, the following primers and probe were designed using the Geneious Prime software version 2023.2.1 (Biomatters Ltd., Auckland, New Zealand):

Forward: 5′-GCAGCTGCGGTAATACATGG;
Reverse: 5′-GAATTCCACTTGCCTCTATCCAA;
Probe: ROX-5′-AGTTCAACGCTTAACGTTGTGATGCTAT-BHQ2.
The presence of chloroplast 16S rDNA was used as an internal control:
Forward: 5′-CGGACGGGAAGTGGTGTTTC;
Reverse: 5′-ACGCGAGCCCCTCCTCGG;
Probe: 6-FAM-5′-CCGTAGGCTGAGGAGCAAAAGGAGGAATC-BHQ1.

The relative load of the pathogen in the samples was related to the level of an internal control using the formula 1000 × 2^(Ct [internal control] − Ct [pathogen])^. For a mutual comparison, the relative phytoplasma load of the samples was normalized to the average phytoplasma load in the original untreated in vitro cultures (set as 1). The obtained data were processed using MS Excel. A *t*-test was used in order to determine the statistical significance between the outputs of various treatment protocols.

## 5. Conclusions

Based on the results of our experiments, the combination of in vitro chemotherapy followed by the in vitro propagation of phytoplasma-free shoots seems to be a promising approach for suppressing AP phytoplasma infections in apples, provided that a balance between the antibiotic efficiency and phytotoxicity is found for the treated plant species or specific cultivar/genotype.

When performing the in vitro sanitation of a systemic internal pathogen such as phytoplasmas, we recommend avoiding the prolonged subculturing of the infected explants prior to the initiation of therapy, as phytoplasma particles may gradually accumulate in the tissues of untreated explants in the course of subcultures.

The next step will be the in vitro rooting of the sanitized culture, followed by an ex vitro transfer. The efficiency of clarithromycin therapy will be conclusively confirmed only if the established in vivo plants remain phytoplasma-free, since chemotherapy can only temporarily reduce the concentration of phytoplasma particles below the detection threshold of the PCR diagnostic used. We also plan to test the genetic stability and observe the fruit traits of the resulting candidate apple-plant-propagating material, to rule out somaclonal variability after the in vitro cultivation phase or unwanted mutations induced by the chemotherapy.

We plan to develop the process of AP elimination and include a pre-treatment with heat to reduce the presence of the pathogen in initial plants. We also envisage the application of the described sanitation procedures to a wider spectrum of apple genetic resources maintained in our institutional gene bank.

## Figures and Tables

**Table 1:** PCR detection of phytoplasmas before and after 1 month of clarithromycin treatment. Average phytoplasma load in initial untreated in vitro cultures was set to 1.

Sample *	DNA Internal Control Ct	AP Phytoplasma Ct	Relative Conc. of Phytopl. DNA
in vivo—mother plant	11.09	15.09	1.9593
in vitro control (1)	11.04	15.95	1.0427
in vitro control (2)	10.48	15.79	0.7902
in vitro control (3)	10.45	15.34	1.0573
in vitro control (4)	12.04	16.86	1.1098
cla10 (1)	10.39	19.89	0.0433
cla10 (2)	10.26	21.49	0.0131
cla10 (3)	10.59	19.69	0.0571
cla10 (4)	9.57	20.76	0.0134
cla10 (5)	11.97	20.64	0.0770
cla10 (6)	10.54	18.84	0.0995
cla10 (7)	10.45	20.46	0.0304
cla10 (8)	9.71	19.73	0.0302
cla10 (9)	10.55	19.92	0.0474
cla10 (10)	10.60	19.51	0.0652
cla10 (11)	10.74	19.78	0.0596
cla10 (12)	10.29	22.85	0.0052
cla10 (13)	9.89	25.19	0.0008
cla10 (14)	10.41	18.03	0.1594
cla10 (15)	10.39	19.79	0.0464
cla20 (1)	10.74	20.97	0.0261
cla20 (2)	10.41	33.99	2.5 × 10^−6^
cla20 (3)	9.74	31.54	8.6 × 10^−6^
cla20 (4)	10.21	19.85	0.0393
cla20 (5)	9.41	21.50	0.0072
cla20 (6)	10.06	22.61	0.0052
cla20 (7)	9.74	20.53	0.0177
cla20 (8)	9.78	24.70	0.0010
cla20 (9)	9.75	21.37	0.0100
cla20 (10)	10.06	–	negative
cla20 (11)	9.82	24.25	0.0014
cla20 (12)	9.87	23.27	0.0029
cla20 (13)	9.92	30.81	1.6 × 10^−5^
cla20 (14)	11.19	23.17	0.0078
cla40 (1)	10.05	24.13	0.0018
cla40 (2)	10.41	22.60	0.0067
cla40 (3)	10.01	34.61	1.2 × 10^−6^

* cla10 = medium with 10 mg/L; cla20 = medium with 20 mg/L; cla40 = medium with 40 mg/L of clarithromycin.

**Table 2:** PCR detection of phytoplasmas 18 months after initial tests. Untreated controls were compared to the material propagated from the phytoplasma-free mericlone cla20 (10). The DNA concentration was related to the average value detected in the initial in vitro material.

Sample	DNA Internal Control Ct	AP Phytoplasma Ct	Relative Conc. of Phytopl. DNA
untreated culture (1)	13.55	17.01	2.8487
untreated culture (2)	13.37	17.01	2.5146
untreated culture (3)	12.76	16.52	2.3139
untreated culture (4)	12.66	16.64	1.9866
cla20 (10)	12.27	–	negative

## Data Availability

The data are available upon request.
